# Prof. Herrick’s Valedictory Address to Graduating Class in Rush Medical College

**Published:** 1851-03

**Authors:** 


### ARTICLE IV.

### PROFESSOR HERRICK'S VALEDICTORY ADDRESS TO THE GRADUATING CLASS IN RUSH MEDICAL COLLEGE.

We have received a copy of this appropriate and well written lecture, through the attention of the Publishing Committee of the class. As we were present in the crowded assembly that listened to it, we can not only testify to the beauty of its style and sentiment, but also to the happy and effective manner of its delivery.

The claims of medicine are briefly urged, the inconsistencies of pretenders referred to, and the graduates pointed to the rich field open before them for cultivation. In the closing paragraph is a tribute to the memory of N. C. Wells, one of the students whose funeral had been attended the day before, reminding the class that "the wreath about to ornament the brows of many here present, is on this occasion, constituted with twigs of the laurel mingled with those of the cypress!"

